# Early Post-operative CT-Angiography Imaging After EC-IC Bypass Surgery in Moyamoya Patients

**DOI:** 10.3389/fneur.2021.655943

**Published:** 2021-03-25

**Authors:** Helene Hurth, Till-Karsten Hauser, Patrick Haas, Sophie Wang, Annerose Mengel, Marcos Tatagiba, Ulrike Ernemann, Nadia Khan, Constantin Roder

**Affiliations:** ^1^Department of Neurosurgery, University Hospital Tuebingen, Tuebingen, Germany; ^2^Center for Moyamoya and Cerebral Revascularization, University Hospital Tuebingen, Tuebingen, Germany; ^3^Department of Diagnostic and Interventional Neuroradiology, University Hospital Tuebingen, Tuebingen, Germany; ^4^Department of Vascular Neurology, University Hospital Tuebingen, Tuebingen, Germany; ^5^Center for Neurovascular Diseases ZNET, University Hospital Tuebingen, Tuebingen, Germany; ^6^Moyamoya Center, University Children's Hospital Zurich, Zurich, Switzerland

**Keywords:** moyamoya, revascularization, computed tomographic angiography, CTA, magnetic resonance time-of-flight angiography, MR-ToF, EC-IC bypass, neurosurgery

## Abstract

**Objective:** To evaluate the clinical value of early post-operative computed tomographic angiography (CTA) after direct extracranial-intracranial (EC-IC) bypass surgery in moyamoya patients.

**Methods:** A retrospective analysis of all adult moyamoya patients treated at our center from 2013 to 2019 with a direct EC-IC bypass was performed. Early post-operative CTA (within 24 h after surgery) was compared with conventional digital subtraction angiography (DSA) 6–12 months after surgery. If available, magnetic resonance time-of-flight angiography (MR-TOF) was evaluated 3 months and 6–12 months post-operatively as well. Imaging results were analyzed and compared with CTA, MR-TOF and DSA, whereat DSA was used as the final and definite modality to decide on bypass patency.

**Results:** A total of 103 direct EC-IC bypasses in 63 moyamoya patients were analyzed. All inclusion criteria were met in 32 patients (53 direct bypasses). In 84.9% the bypass appeared definitively, in 5.7% uncertainly and in 9.4% not patent according to early post-operative CTA. MR-TOF suggested definitive bypass patency in 86.8% 3 months after surgery and in 93.5% 6–12 months after surgery. DSA 6–12 months post-operatively showed a patency in 98.1% of all bypasses. The positive predictive value (to correctly detect an occluded bypass) on post-operative CTA was 12.5%, the negative predictive value (to correctly detect a patent bypass) was 100% with a sensitivity of 100% and a specificity of 86.5%.

**Conclusion:** Early post-operative CTA has a high predictive value to confirm the patency of a bypass. On the other hand, a high false positive rate of (according to CTA) occluded bypasses after direct EC-IC bypass surgery can be seen. This must be considered critically when initiating possible therapeutic measures.

## Introduction

Surgical revascularization with direct extracranial-intracranial (EC-IC) bypass is the most common therapy in adult moyamoya patients (1, 2). Long-term bypass patency has been shown to be between 88 and 97% in these patients (3–6). The risk of bypass occlusion appears highest during the first week after surgery and declines over time as the bypass matures (3).

Digital subtraction angiography (DSA) is the “gold standard” for the imaging of cerebral vessels (3, 4, 7, 8). However, non-invasive techniques, such as computed tomography angiography (CTA) or time-of-flight magnetic resonance angiography (MR-TOF) can also be used to examine the cerebral vessels with high precision (9–13). CTA has been reported to have a specificity of 88% to 100% and even higher sensitivity for the detection and follow-up of intracranial aneurysms compared to DSA (7, 10, 13). MR-TOF has been shown to have a specificity of up to 94% and sensitivity of 88% in the same context (9, 11, 12). Little is known about the clinical value of early post-operative CTA after EC-IC bypass surgery in moyamoya patients. A small cohort with 11 patients revealed a slightly lower specificity of post-operative CTA compared to DSA but an identical sensitivity regarding early-post-operative bypass patency (8). A comparison of early post-operative CTA and MR-TOF revealed a similar visualization of EC-IC bypasses in moyamoya patients (14).

Aim of this study was to analyze the clinical value of early post-operative CTA compared to long-term results of MR-TOF and DSA regarding the bypass patency in adult moyamoya patients after EC-IC bypass surgery.

## Methods

A single-center retrospective analysis was performed including all consecutive adult moyamoya patients treated at our center between 2013 and 2019. Patients who had received a direct STA-MCA bypass, an early post-operative CTA and a DSA after 6 to 12 months were included. The primary aim of the study was to calculate sensitivity, specificity, positive and negative predictive value of early post-operative CTA. In addition, the association of bypass patency with patients' characteristics was investigated.

The presence of moyamoya angiopathy was proven in all cases angiographically. All patients received pre- and post-operative diagnostic imaging according to a routine protocol (which has been adapted over time) including pre-operative H2 15O PET CT with acetazolamide challenge, early post-operative CTA within 24 h, MR-TOF ~3 months and DSA as well as MR-TOF approximately 6 to 12 months after surgery. Indication for revascularization was based on the results of H2 15O PET CT with acetazolamide challenge in all cases. Inclusion criteria for this study were the availability of early post-operative CTA and DSA after 6 to 12 months post-operatively. Patients with direct bypasses other than STA-MCA were excluded.

Bypass patency was evaluated independently for each imaging modality and each bypass blinded for the longitudinal imaging findings of each patient. Results were categorized as “patent,” “uncertain” or “occluded.” Bypasses were rated as uncertain if only the proximal donor branch was visible but could not be traced to the anastomosis. Bypasses were considered occluded if neither the proximal nor the distal part of the donor vessel could be visualized. Occluded bypasses and those with uncertain patency were reevaluated by a second reviewer according to the same criteria. Divergent results were finally decided in consensus. For statistical processing, uncertain bypasses were classified as non-patent. Additionally, MR-TOF images 3 months and 6 to 12 months after surgery were analyzed if available, following the same criteria as for CTA and DSA. Results of 6 to 12 months post-operative DSA were used as the final criterion for bypass patency. General clinical data was collected from the patients' medical files.

### Statistics

Data acquisition and statistical analyses were performed with IBM® SPSS Statistics 21 (IBM Corporation, Armonk, NY, USA) and Microsoft® Excel 16.16.11 (Microsoft Corporation, Redmond, WA, USA). Due to the exploratory character of the study no a-priori case number calculation was performed. Metric variables were tested for normal distribution using the Shapiro-Wilk test. Normally distributed data was compared by means of *t*-tests for dependent or independent variables, as applicable. Nominally scaled data was analyzed with the Chi-Square or Fisher's-Exact test. *P*-values < 0.05 were considered as significant. The confidence interval was assumed to be 95%. Sensitivity (truly occluded/occluded in imaging modality), specificity (truly patent/patent in imaging modality), negative (truly occluded/truly and falsely occluded in imaging modality) and positive (truly patent/truly and falsely patent in imaging modality) predictive values were calculated for CTA and MR-TOF.

### CTA Imaging

CT images were acquired on a 128-slice CT (Somatom Definition AS+, Siemens Healthcare AG, Forchheim, Germany). CT angiography was performed using bolus tracking after intravenous injection of 50 ml iodinated contrast agent (Imeron®, 400 mg iodine/ml, Bracco Imaging, Konstanz, Germany) followed by a 60-ml saline chaser at a flow rate of 4 ml/s. For the CTA the following scanning parameters were used: automatic tube current modulation (CARE Dose 4D, Siemens Healthcare AG, Forchheim, Germany) with a reference mAs setting of 120, Care kV with a reference setting of 120 kV, 0.75 mm collimation, 0.3 s rotation time, 0.55 spiral pitch factor. Axial MPRs were reconstructed from the raw data with a FOV of 240 mm, matrix size 512 × 512 and a slice thickness of 1.5 mm using the reconstruction kernel I26f in conjunction with advanced modeled iterative reconstruction (ADMIRE) software (Siemens Healthcare AG) strength 2.

### MR-TOF Imaging

All follow-up MR scans at our hospital were done on a clinical 3T Scanner (Magnetom Skyra, Siemens Healthcare, Erlangen, Germany) using a 3D TOF-MRA sequence with the following parameters: TE 3.73 ms, TR 22 ms, flip angle 18°, FoV 177 × 210 mm, slice thickness 0.60 mm, pixel spacing 0.23 mm. The acquisition time was 3:36 min.

### Ethics

Ethical approval for this study was obtained from the Ethics Committee at the Medical Faculty of the University of Tuebingen (909/2020BO2).

## Results

A total of 103 direct EC-IC bypasses in 63 moyamoya patients were analyzed. Of those, 32 patients with 53 bypasses met the inclusion criteria and were reviewed in detail. The main reason for exclusion from the analysis was a non-available CTA or DSA in the defined time period. Four bypasses other than STA-MCA were excluded. The mean patient age at the time of surgery was 41.1 years (sd = 15.5) with an age range of 17 to 67 years. 78.1% (*n* = 25) were female and 21.9% (*n* = 7) were male (Table 1). Twenty-eight bypasses were on the right, 25 on the left side. Twenty-one patients (65.6%) received bilateral bypasses.

**Table 1:** Characteristics of all included patients.

**Patient characteristics**		
Gender % (*N*) of patients	Female	Male
	75.8% (25)	24.2% (8)
Unilateral/bilateral angiopathy % (*N*) of patients	Unilateral	Bilateral
	34.4% (11)	65.6% (21)

Intraoperatively, the bypasses were described as patent (proven by duplex sonography and ICG) in all cases at the end of surgery. In four cases the blood flow of the donor vessel was limited after preparation caused by vasospasm or thrombosis. Blood flow was restored successfully in all four cases by local medical therapy (heparin, papaverine) before placing the anastomosis. The intraoperative blood flow was described as promptly in 88.7% (*n* = 47) and delayed in 11.3% (*n* = 6) of the cases (Table 2).

**Table 2:** Bypass characteristics. Cross table showing percentage and number of bypasses in which an intraoperative temporary thrombosis (which was resolved before suturing the anastomosis) of the donor vessel was observed and bypass blood flow at the end of surgery.

**Bypass characteristics**		**Intraoperative blood flow**			
**% of bypasses (*N*)**		**Promptly**	**Delayed**	**Occluded**	**Total**
Intraoperative thrombosis	Yes	3.8% (2)	3.8% (2)	0% (0)	**7.5%** (4)
	No	84.9% (45)	7.5% (4)	0% (0)	**92.5%** (49)
	Total	**88.7%** (47)	**11.3%** (6)	**0%** (0)	**100%** (53)

*Intraoperative blood flow is described as written by the surgeon (NK and CR) in the surgical report based on visual inspection only without invasive measurement as promptly, delayed or occluded*.

### Bypass Patency According to CTA

**Figure 1:**
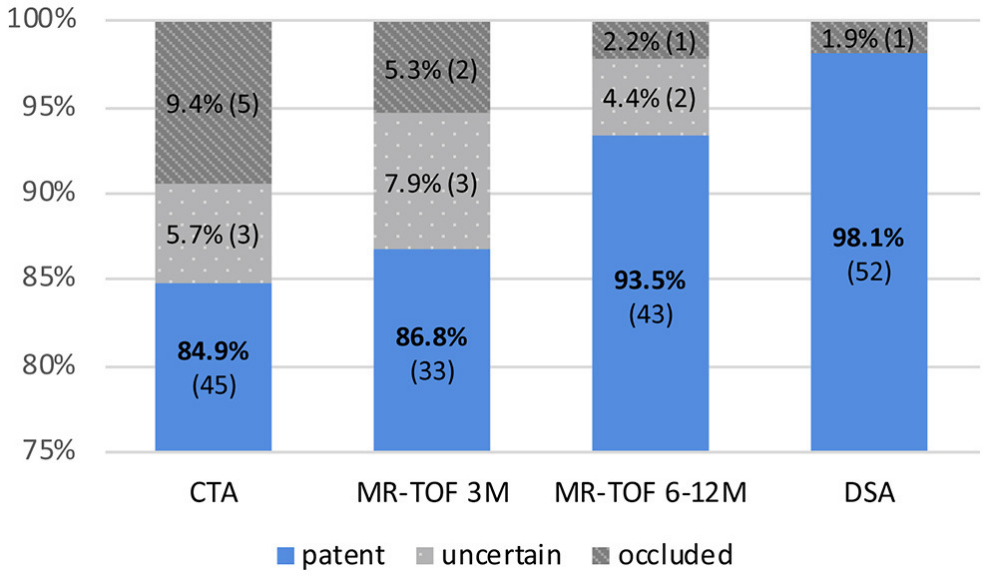
Percentage and number of bypasses shown as “patent,” “uncertain,” and “occluded” in CTA post-operatively, MR-TOF after 3 months (3M), MR-TOF after 6 to 12 months (6–12 M) and DSA after 6 to 12 months.

**Figure 2:**
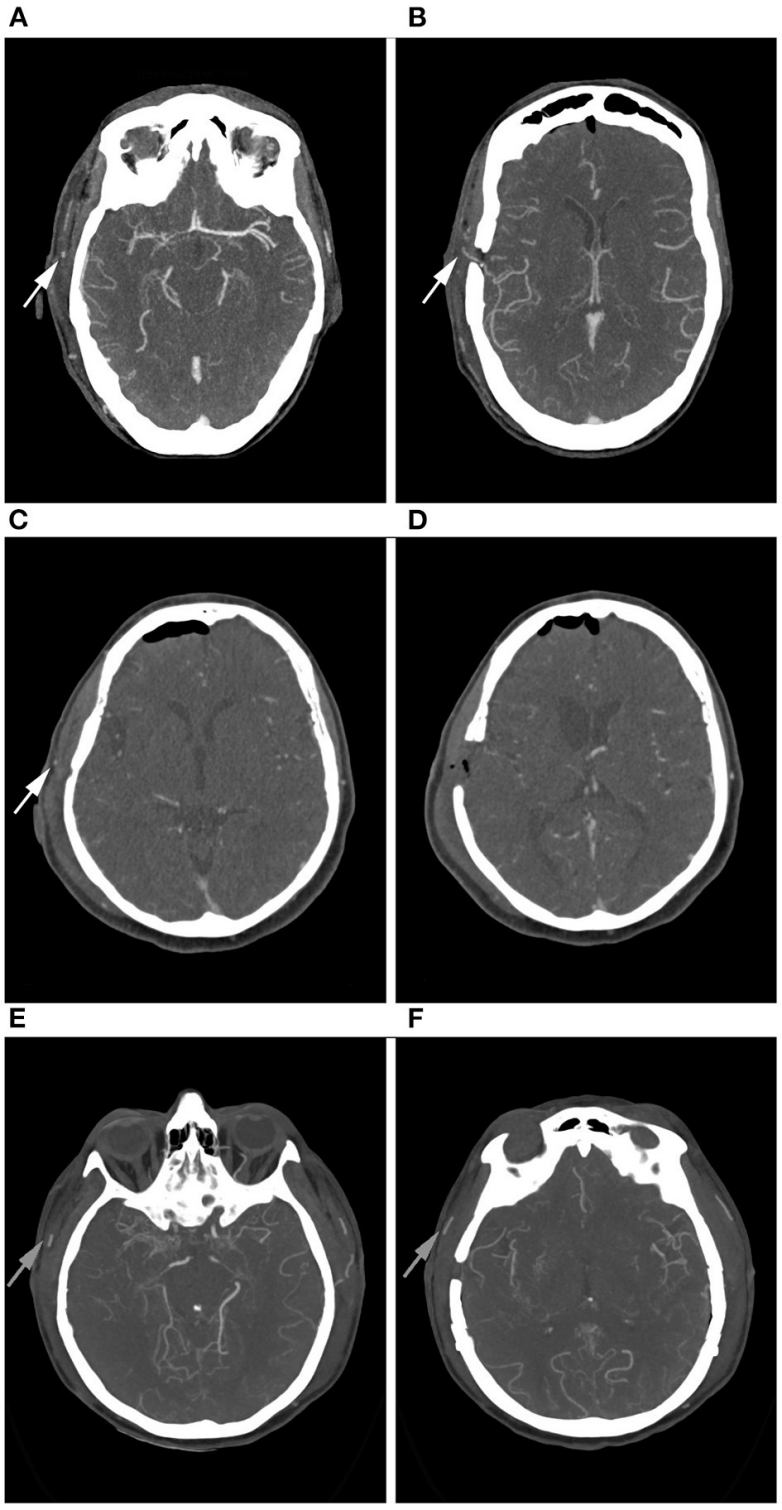
Example of right-sided STA-MCA bypasses in CTA. **(A,B)**: Bypass is patent. The proximal **(A)** and the distal **(B)** parietal branch of the STA are visible (arrows). **(C,D)**: Bypass of uncertain patency. Only the proximal branch of the STA is visible (**C**, arrow), the distal branch is not clearly contrasted **(D)**. **(E,F)**: Bypass appears occluded. Neither the proximal **(E)** nor the distal **(F)** parietal branch of the STA are contrasted. Good contrast enhancement of the frontal branch of the STA (arrows).

In 84.9% (*n* = 45) of all cases the bypass was clearly patent in the early post-operative CTA. In 5.7% (*n* = 3) patency was uncertain and in 9.4% (*n* = 5) CTA was suspicious for an occluded bypass (Figure 1). Examples for patent, uncertainly patent and suspiciously occluded bypasses are shown in Figure 2.

### Bypass Patency According to MR-TOF

MR-TOF after 3 months was available for 38, after 6 to 12 months for 46 bypasses. Three-month post-operative MR-TOF showed the bypass to be clearly patent in 86.8% (*n* = 33), uncertainly patent in 7.9% (*n* = 3) and suspicious for occlusion in 5.3% (*n* = 2).

MR-TOF 6 to 12 months after surgery showed a clearly patent bypass in 93.5% (*n* = 43), an uncertain patency in 4.3% (*n* = 2) and occlusion in 2.2% (*n* = 1) of all cases.

### Bypass Patency According to DSA

**Figure 3:**
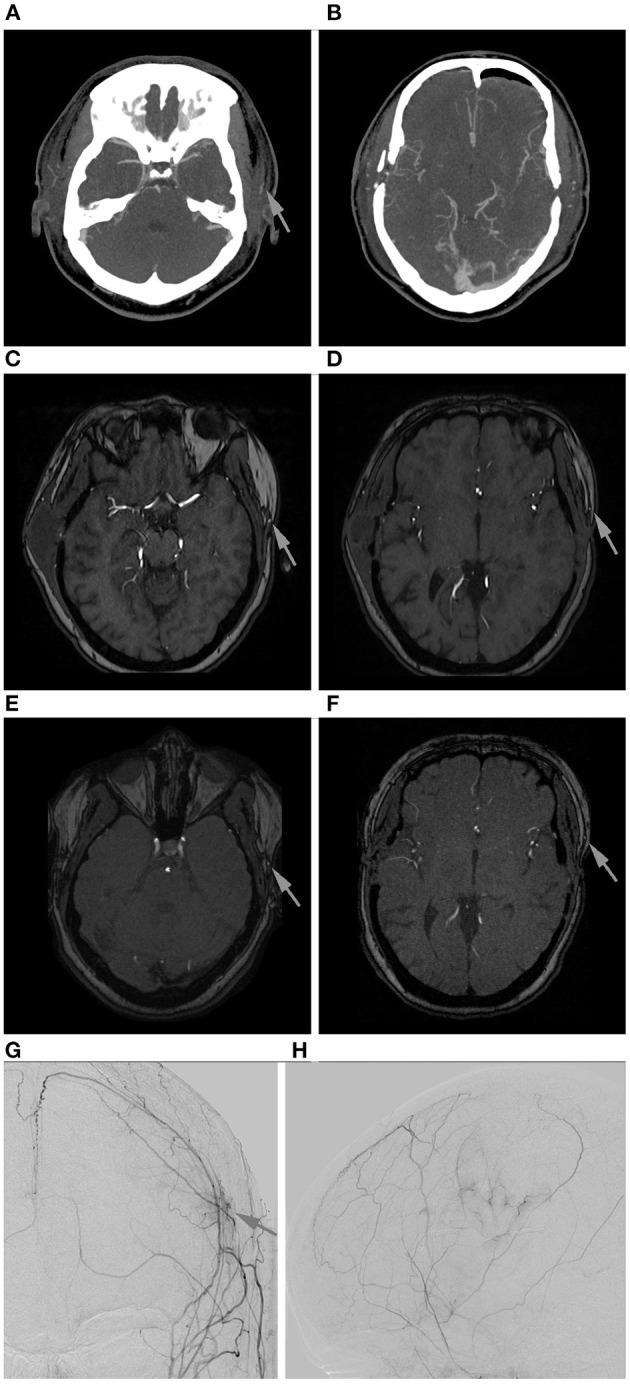
Follow-up of a left-sided STA-MCA bypass shown to be occluded in DSA. **(A,B)**: Early post-operative CTA. Only the frontal branch of the STA is contrasted (arrow). **(C,D)**: MR-TOF after 3 months. Only the frontal branch of the STA is contrasted (arrows). **(E,F)**: MR-TOF after 6 months. Only the frontal branch of the STA is contrasted (arrows). **(G,H)**: Angiogram of the external carotid artery anterior-posterior **(G)** and lateral **(H)** with only little cortical perfusion at the site of revascularization (arrow; vessel sprouting caused by indirect revascularization with additional EGPS (MCA territory) and in the ACA territory (spontaneous collateralization caused by the middle meningeal artery).

The result of DSA 6 to 12 months after surgery was the final criterion for bypass patency and showed a patency rate of 98.1% (*n* = 52) of all direct revascularizations. One STA-MCA bypass was occluded at that time (1.9%), which was concordant with the previous imaging of this patient (see Figure 3). In this single case, intraoperatively impaired blood flow of the donor vessel was seen caused by local thrombosis which, however, was restored to normal blood flow after medical therapy with local intraluminal heparin.

There was no significant difference in bypass patency regarding gender, age or side of the hemisphere for the post-operative CTA, MR-TOF after 3 months or MR-TOF after 6 to 12 months [CTA: gender—Fisher's exact, *p* = 0.51; age − 95%-CI(−11.28, 12.78), *t*(51) = 0.13, *p* = 0.90; side—Fisher's exact, *p* = 86; MR-TOF after 3 months: gender—Fisher's exact, *p* = 1; age − 95%-CI(−21.96, 8.73), *t*(36) = −0.87, *p* = 0.39; side—Fisher's exact, *p* = 0.78; MR-TOF after 6 to 12 months: gender—Fisher's exact, *p* = 1; age–95%-CI(−26.06, 12.51), *t*(44) = −0.71, *p* = 0.48; side—Fisher's exact, *p* = 0.73].

The overall sensitivity to detect an angiographically proven, truly occluded bypass was 100% for CTA and MR-TOF examinations. The specificity of detecting an angiographically proven patent bypass was 86.5% for post-operative CTA, 89.2% for MR-TOF after 3 months and 95.6% for MR-TOF after 6 to 12 months.

The positive predictive value (as the probability for a bypass to be truly occluded when shown uncertainly or non-patent in the post-operative CTA) was 12.5%. For MR-TOF the positive predictive value was 20% after 3 months and 33% after 6 to 12 months. The negative predictive value (the probability for a bypass to be truly patent in DSA when shown patent in CTA or MR-TOF) was 100%.

## Discussion

In this study we were able to show that the post-operative CTA after well-indicated EC-IC bypass surgery is highly reliable to confirm the patency of a bypass compared to the 6 to 12-month post-operative DSA. On the other hand, a relatively high false positive rate of bypasses appearing as occluded in early post-operative CTA was seen.

Overall definite bypass patency after 6 to 12 months was excellent with 98.1% and, therefore, slightly higher than reported in the literature (3, 4). One bypass which was confirmed to be occluded in the follow-up DSA was already displayed as non-patent in the post-operative CTA. This assumed very early occlusion of the bypass supports data from earlier studies that showed, that risk of bypass occlusion is highest during the first post-operative week (3). As we always place an additional indirect bypass by encephalo-galea-periost-synangiosis (EGPS) on the brain when doing a direct revascularization, the patient's body can build collaterals through the indirect bypass, as also seen in this case (Figures 3G,H). This, however, takes many months until the revascularization has reached relevant blood flow.

Our data show a sensitivity of 100% for the post-operative CTA as well as for MR-TOF on follow-up examinations with a slightly lower specificity. This is consistent with data from smaller cohorts investigating EC-IC bypasses by means of CTA (8, 15). However, sensitivity rates might be falsely high due to the low number of occluded bypasses in our cohort. We found a continuous increase of specificity from 86.5% in the direct post-operative examination to 95.6% after 6 to 12 months. Although different imaging modalities are compared in this case (CTA and MR-TOF) earlier examinations showed similar results on bypass visualization regarding its patency for both examination methods (14). Also, the MR-TOF specificity rate of 95.6% after 6 to 12 months almost equals findings from Park et al. who reported a specificity for CTA compared to DSA of 95.5% regarding bypass patency. In that study CTA was performed 5 to 9 months after surgery (15), which is comparable to our data.

A possible explanation for an initially higher rate of an incorrectly implied bypass occlusion is the low-flow nature of single-branch STA-MCA bypasses (16, 17). Since the time-point of the CT scan after application of the contrast agent is determined to optimally display the intracranial arteries (based on the common carotid artery), time of contrast wash-in might be delayed for the STA with a slow flow in the early post-operative phase. This, however, improves over time as the blood flow through the bypass usually increases over time depending on the intracranial blood demand (3). Therefore, a higher residual anterograde blood flow at the site of the anastomosis competing with the applied retrograde STA-MCA perfusion (2) might lead to a slower blood flow during the early post-operative period leading to false positive results on CTA imaging.

In some cases, initially uncertainly patent bypasses were displayed patent in the DSA after 6 to 12 months but didn't mature as much over time as expected. This might be a consequence of a lower intracranial blood demand than pre-operatively estimated despite extended pre-operative examinations (18).

The question arises, which clinical consequences should be derived from these results. In our institution, usually no emergency revision surgeries are performed after detecting a possibly occluded bypass on CTA. Adequate volume management should be maintained in the early post-operative phase in all patients. Extended continuous blood-pressure monitoring and intravascular volume supplementation might be considered. Also, indirect revascularization might develop even if direct revascularization fails as we usually place an EGPS in addition to direct EC-IC bypass surgery on the brain to possibly enhance indirect vessel sprouting, if needed.

In conclusion, our findings do not reveal any substantial benefits of early post-operative CTA due to a high false-positive rate for bypass occlusion. Considering the additional costs and exposure to radiation routine early post-operative CTA might not be recommended in the early post-operative course.

## Limitations

This is a retrospective single-center study in moyamoya patients. Imaging was only rated by one person as patent bypasses are clearly identifiable. For uncertain or possibly occluded bypasses a second rater was asked for final consensus. We routinely do not use ultrasound examinations in the direct post-operative phase, which however might be a good tool to monitor bypass-patency but has several limitations in the early post-operative phase caused by local swelling and the wound healing. Only one bypass was seen to be truly occluded after 6 to 12 months. Therefore, sensitivity rates in this study might appear higher due to the low number of occluded bypasses. A relatively high number of overall drop-outs is based on the fact that the routine imaging protocol developed over time and, therefore, not all imaging was available for each patient at all time-points.

## Conclusions

Our analysis shows a high reliability of the post-operative CTA to confirm the patency of a bypass compared to the 1-year post-operative DSA. On the other hand, a relatively high false positive rate of bypasses appearing as occluded after direct EC-IC bypass surgery is shown in early post-operative CTA. This should be critically considered when initiating early surgical revision or extended blood pressure and intravascular volume-management based on CTA. Routine early post-operative CTA might not be recommended for the evaluation of bypass patency in the early post-operative course.

## Data Availability Statement

The raw data supporting the conclusions of this article will be made available by the authors, without undue reservation.

## Ethics Statement

The studies involving human participants were reviewed and approved by Ethics Committee of the Medical Faculty of the University of Tuebingen (909/2020BO2). Written informed consent for participation was not required for this study in accordance with the national legislation and the institutional requirements.

## Author Contributions

Data analysis was performed by HH and CR. The first draft of the manuscript was written by HH and all authors commented on previous versions of the manuscript. All authors contributed to the study conception, design, material preparation, and data collection. All authors read and approved the final manuscript.

## Conflict of Interest

The authors declare that the research was conducted in the absence of any commercial or financial relationships that could be construed as a potential conflict of interest.

## References

[1] Scott RM Smith ER. Moyamoya disease and moyamoya syndrome. N Engl J Med. (2009) 360:1226–37. 10.1056/NEJMra0804622 19297575

[2] Fujimura M Bang OY Kim JS. Moyamoya disease. Front Neurol Neurosci. (2016) 40:204–220. 10.1159/000448314 27960175

[3] Yoon S Burkhardt J-K Lawton MT. Long-term patency in cerebral revascularization surgery: an analysis of a consecutive series of 430 bypasses. J Neurosurg. (2019) 131:80–7. 10.3171/2018.3.JNS172158 30141754

[4] Ge P Ye X Liu X Deng X Wang J Wang R. Angiographic outcomes of direct and combined bypass surgery in moyamoya disease. Front Neurol. (2019) 10:1267. 10.3389/fneur.2019.01267 31849825 PMC6903770

[5] Aboukais R Verbraeken B Leclerc X Gautier C Henon H Vermandel M. Superficial temporal artery-middle cerebral artery anastomosis patency correlates with cerebrovascular reserve in adult moyamoya syndrome patients. Neurochirurgie. (2019) 65:146–51. 10.1016/j.neuchi.2019.05.001 31185229

[6] The EC/IC Bypass Study Group*. Failure of extracranial–intracranial arterial bypass to reduce the risk of ischemic stroke: results of an international randomized trial. N Engl J Med. (1985) 313:1191–200. 10.1056/NEJM198511073131904 2865674

[7] Karamessini MT Kagadis GC Petsas T Karnabatidis D Konstantinou D Sakellaropoulos GC. CT angiography with three-dimensional techniques for the early diagnosis of intracranial aneurysms. Comparison with intra-arterial DSA and the surgical findings. Eur J Radiol. (2004) 49:212–23. 10.1016/S0720-048X(03)00173-6 14962650

[8] Teksam M McKinney A Truwit CL. Multi-slice CT angiography in evaluation of extracranial–intracranial bypass. Eur J Radiol. (2004) 52:217–20. 10.1016/j.ejrad.2003.12.003 15544897

[9] Deutschmann HA Augustin M Simbrunner J Unger B Schoellnast H Fritz GA. Diagnostic accuracy of 3D time-of-flight MR angiography compared with digital subtraction angiography for follow-up of coiled intracranial aneurysms: influence of aneurysm size. AJNR Am J Neuroradiol. (2007) 28:628–34. 10.3109/02688690309177971 17416811 PMC7977342

[10] Dehdashti AR Rufenacht DA Delavelle J Reverdin A de Tribolet N. Therapeutic decision and management of aneurysmal subarachnoid haemorrhage based on computed tomographic angiography. Br J Neurosurg. (2003) 17:46–53. 12779201

[11] Ahmed SU Mocco J Zhang X Kelly M Doshi A Nael K. MRA versus DSA for the follow-up imaging of intracranial aneurysms treated using endovascular techniques: a meta-analysis. J NeuroIntervent Surg. (2019) 11:1009–14. 10.1136/neurintsurg-2019-014936 31048457

[12] van Amerongen MJ Boogaarts HD de Vries J Verbeek ALM Meijer FJA Prokop M. MRA versus DSA for follow-up of coiled intracranial aneurysms: a meta-analysis. Am J Neuroradiol. (2014) 35:1655–61. 10.3174/ajnr.A3700 24008171 PMC7966263

[13] Villablanca JP Jahan R Hooshi P Lim S Duckwiler G Patel A. Detection and characterization of very small cerebral aneurysms by using 2D and 3D helical CT angiography. AJNR Am J Neuroradiol. (2002) 23:1187–98. 12169479 PMC8185733

[14] Chen Q Qi R Cheng X Zhou C Luo S Ni L. Assessment of extracranial-intracranial bypass in Moyamoya disease using 3T time-of-flight MR angiography: comparison with CT angiography. Vasa. (2014) 43:278–83. 10.1024/0301-1526/a000363 25007906

[15] Park JC Kim JE Kang H-S Sohn C-H Lee DS Oh CW. CT Perfusion with angiography as a substitute for both conventional digital subtraction angiography and acetazolamide-challenged SPECT in the follow-up of postbypass patients. Cerebrovasc Dis. (2010) 30:547–55. 10.1159/000319026 20948198

[16] Ramanathan D Temkin N Kim LJ Ghodke B Sekhar LN. Cerebral bypasses for complex aneurysms and tumors. Neurosurgery. (2012) 70:1442–57. 10.1227/NEU.0b013e31824c046f 22278357

[17] Cherian J Srinivasan V Kan P Duckworth EA. Double-barrel superficial temporal artery-middle cerebral artery bypass: can it be considered “high-flow?” *Oper Neurosurg*. (2018) 14:288–94. 10.1093/ons/opx119 28961997

[18] Roder C Bürkle E Ebner FH Tatagiba M Ernemann U Buck A. Estimation of severity of moyamoya disease with [15O]water-positron emission tomography compared with magnetic resonance imaging and angiography. World Neurosurg. (2018) 117:e75–e81. 10.1016/j.wneu.2018.05.163 29886291

